# Analysis of genetic factors associated with fatty liver disease‐related hepatocellular carcinoma

**DOI:** 10.1002/cam4.6410

**Published:** 2023-08-29

**Authors:** Tomomi Kogiso, Yuri Ogasawara, Kentaro Horiuchi, Makiko Taniai, Katsutoshi Tokushige

**Affiliations:** ^1^ Department of Internal Medicine Institute of Gastroenterology, Tokyo Women's Medical University Tokyo Japan

**Keywords:** 17‐beta dehydrogenase 13, fatty liver disease, hepatocellular carcinoma, patatin‐like phospholipase 3

## Abstract

**Aim:**

Single‐nucleotide polymorphisms (SNPs) in *PNPLA3* and hydroxysteroid 17‐beta dehydrogenase 13 (*HSD17B13*) genes are associated with fatty liver disease (FLD) progression and carcinogenesis. In the present study, we evaluated the characteristics of Japanese FLD patients according to *HSD17B13* polymorphisms.

**Methods:**

We enrolled 402 patients who were clinically and pathologically diagnosed with FLD (alcoholic: 63 cases, nonalcoholic: 339 cases) at our hospital in 1990–2018 (228 males; median age: 54.9 [14.6–83.6] years). FLD patients with *HSD17B13* A/A (212 cases) and others (A/AA or AA/AA; 190 cases) were compared.

**Results:**

Compared to patients with *HSD17B13* A/A and others, those with the A/A genotype showed increased incidence of hepatocellular carcinoma (HCC) (A/A vs. others; 18.4% vs. 9.5%, *p* = 0.01), cardiovascular diseases (14.2% vs. 4.2%, *p* < 0.01), and hypertension (56.6% vs. 47.4%, *p* = 0.06). In patients without A/A, the HCC incidence was significantly reduced in those with alcohol‐related FLD, fibrosis‐4 index <2.67, and the *PNPLA3* CC genotype; however, there was no significant difference in nonalcoholic‐FLD. Patients without *HSD17B13* A/A showed severe steatosis (77% vs. 88.6%, *p* < 0.01). New HCC developed in 11 cases and the 5–year incidence rate of HCC was 3.3% in patients with both *PNPLA3* GG/GC and *HSD17B13* A/A, which was significantly higher than the rate for those with other SNP profiles (0.6%, *p* = 0.03).

**Conclusions:**

Inhibiting *HSD17B13* activity may prevent HCC development, particularly in alcohol‐related FLD and low‐risk patients. Therefore, combinations of SNPs and other risk factors can be used for screening FLD–HCC.

## Graphical abstract

Patients with the HSD17B13 A/A genotype showed increased incidence of hepatocellular carcinoma, cardiovascular diseases, and hypertension. In patients without HSD17B13 A/A, the HCC incidence was significantly reduced in those with alcohol‐related FLD, fibrosis‐4 index <2.67, and the PNPLA3 CC genotype. In patients with both PNPLA3 GG/GC and HSD17B13 A/A, the HCC incidence was significantly increased.

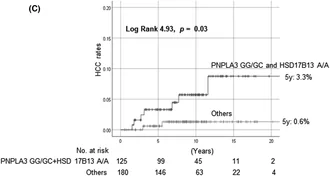

## 1 INTRODUCTION

Nonalcoholic fatty liver disease (NAFLD), nonalcoholic steatohepatitis (NASH), and alcohol‐related liver disease (ALD) are chronic liver diseases that have become increasingly common causes of hepatocellular carcinoma (HCC) in Japan, likely due to cure of viral hepatitis.^1^ These lifestyle‐related diseases are associated with the development of HCC, particularly in patients with type 2 diabetes mellitus.^2^ In a nationwide survey conducted in Japan, the annual incidence of HCC was 0.11%. In patients with diabetes, the risk factors of HCC were high fibrosis‐4 (FIB‐4) index, male sex, hypertension, low hemoglobin _A1c_ and albumin levels, and high body mass index (BMI), and gamma‐glutamyl transpeptidase levels.^2^

Genome‐wide association studies have demonstrated that the patatin‐like phospholipase 3 (*PNPLA3*) GG single‐nucleotide polymorphism (SNP; rs738409 [G] encoding I148M) increases the risk of NAFLD development and HCC.^3^ The prevalence of the *PNPLA3* risk allele is higher in Japan than in Western countries (21% and 5%, respectively).^4^, ^5^, ^6^ In our previous study, we identified an association between the *PNPLA3* GG genotype and fibrosis initiation and development in NAFLD patients.^7^ Additionally, mutations in hydroxysteroid 17‐beta dehydrogenase 13 (*HSD17B13*), which encodes hepatic lipid droplet protein, are involved in various metabolic processes.^8^, ^9^ Loss‐of‐function mutations in the human *HSD17B13* gene may confer a strong protective effect against liver injury, inflammation, fibrosis, and HCC onset.^9^, ^10^

In the present study, we investigated the risk of HCC development according to the *PNPLA3* and *HSD17B13* SNPs in FLD patients. Additionally, we assessed the associations of HCC development with liver fibrosis and metabolic diseases.

## 2 METHODS

### 2.1 Study participants and design

This observational single‐center study enrolled 402 FLD Japanese patients (339 NAFLD and 63 ALD) diagnosed between 1990 and 2018 at Tokyo Women's Medical University Hospital. The patients were not deliberately chosen and it was a bias‐free population. NAFLD was diagnosed according to clinical practice guidelines; 298 patients had biopsy‐confirmed NAFLD.^11^ We confirmed the diagnoses of all study participants and excluded patients who did not fulfill the diagnostic criteria. The pathological stage of NAFLD was evaluated using the classification system of Brunt et al.^12^ Advanced fibrosis was defined as fibrosis stage ≥3. For ALD, ethanol intake was defined as >60 g/day.^13^

The diagnosis of diabetes was based on the American Diabetes Association criteria^14^ or the use of anti‐diabetic agents. Dyslipidemia and hypertension were diagnosed on the basis of the diagnostic criteria for metabolic syndrome^15^, ^16^ or medication use. Laboratory data were collected at the time of liver biopsy or hospital admission.

This study was conducted in accordance with the Declaration of Helsinki and the ethical guidelines of the Tokyo Women's Medical University Hospital (Tokyo, Japan). The Institutional Review Board of Tokyo Women's Medical University Hospital approved the study protocol. Informed consent was obtained from the study participants.

### 2.2 Analysis of genetic polymorphisms

*PNPLA3* and *HSD17B13* SNPs were evaluated in 387 and 402 patients, respectively. Genomic DNA was extracted from whole blood. The SNPs rs738409 (*PNPLA3*) and rs72613567 (*HSD17B13*) were assessed in patients using the MassARRAY® system (Agena Bioscience) with iPLEX chemistry, which combines mass spectrometry and polymerase chain reaction. The primer sequences (5′–3′) used in this study were designed for the specific sites in each gene (rs738409 F: ACGTTGGATGAGAGAAAGCCGACTTACCAC and rs738409 R: ACGTTGGATGTCACAGGCCTTGGTATGTTC. rs72613567 F: GCTCTATTGGTGTTTTAGTATTTGGGTGTT and rs72613567 R: AGAAGTCTGATAGATGGAATACTTACCAATAAGAA).

### 2.3 Statistical analysis

Data are presented as medians and ranges. We compared the variables between patients with *HSD17B13* A/A vs. A/AA or AA/AA. Differences were assessed by the Kruskal‐Wallis test, Mann–Whitney *U*‐test, or χ^2^ test using SPSS software (IBM Corp.). *P*‐values <0.05 were considered indicative of statistical significance. We performed multivariate analyses of dataset 1 to explore the potential risk factors of HCC, including age, sex, complications of diabetes mellitus, dyslipidemia, hypertension, BMI, pathological fibrosis, inflammation, and steatosis, and SNPs of *PNPLA3* and *HSD17B13*. The multivariate analyses were repeated for dataset 2 to assess the potential risk factors of age, sex, complications of diabetes mellitus, dyslipidemia, hypertension, BMI, FIB‐4 index, and SNPs of *PNPLA3* and *HSD17B13*. Furthermore, the risk of new onset of HCC was evaluated by Cox proportional hazard model for dataset 2. The odds ratios (ORs) and 95% confidence intervals (CIs) were calculated for the HCC risk.

Cumulative curves for new‐onset HCC were constructed using the Kaplan–Meier method. The incidence of HCC was compared using the log‐rank test.

## 3 RESULTS

### 3.1 Demographic characteristics of FLD patients with HSD polymorphism

This study included 402 Japanese patients with FLD who were diagnosed between 1990 and 2018 (63 with ALD and 339 with NAFLD). The diagnosis was confirmed on biopsy in 298 of these patients. We compared the clinical features of FLD patients according to the *HSD17B13* polymorphisms (Table 1). Compared to patients with *HSD17B13* wild–type A/A (212 cases) and variants (A/AA or AA/AA, 190 cases), there were no significant differences in the age, sex distribution, BMI, ALD, or prevalence of diabetes or dyslipidemia. Patients with *HSD17B13* variants had significantly lower rates of HCC complications (A/A vs. variants; 39 [18.4%] vs. 18 [9.5%], *p* = 0.01) and hypertension (120 [56.6%] vs. 90 [47.4%], *p* = 0.06). The incidence of cardiovascular disease (CVD) was also reduced in patients with *HSD17B13* variants (30 [14.2%] vs. 8 [4.2%], *p* < 0.01). Patients with *HSD17B13* variants had a higher triglyceride level than those without the variants (133 vs. 143 mg/dL, *p* = 0.08). There were no significant differences in the other laboratory findings, including the FIB‐4 index, among patients with various *HSD17B13* polymorphisms.

**TABLE 1:** Characteristics of patients with NAFLD according to *HSD17B13* polymorphism.

Variable	Total (*N* = 402)	*HSD17B13* A/A (*n* = 212)	*HSD17B13* A/AA, AA/AA (*n* = 190)	*p*‐value
Age (years)	55 (15–84)	56 (15–84)	54 (16–80)	0.14
Male sex, *n* (%)	228 (56.7%)	126 (59.4%)	102 (53.7%)	0.25
BMI, kg/m^2^	26.6 (12.8–46.0)	26.4 (16.1–45.0)	26.8 (12.8–46.0)	0.70
Diabetes mellitus (%)	191 (47.5%)	102 (48.1%)	89 (46.8%)	0.80
Dyslipidemia (%)	249 (61.9%)	138 (65.1%)	111 (58.4%)	0.17
Hypertension (%)	210 (52.2%)	120 (56.6%)	90 (47.4%)	0.06
Alcoholic/nonalcoholic FLD	63/339	39/173	24/166	0.11
HCC (%)	57 (14.2%)	39 (18.4%)	18 (9.5%)	0.01
CVD events (%)	38 (9.5%)	30 (14.2%)	8 (4.2%)	<0.01
Extrahepatic malignancies (%)	44 (10.9%)	25 (11.8%)	19 (10.0%)	0.57
Laboratory data				
Albumin, g/dL	4.3 (1.6–5.5)	4.2 (1.7–5.5)	4.3 (1.6–5.4)	0.24
Total bilirubin, mg/dL	0.7 (0.2–43.5)	0.8 (0.2–8.1)	0.7 (0.3–43.5)	0.13
Aspartate aminotransferase, U/L	47 (12–2609)	46 (13–1024)	49 (12–2609)	0.37
Alanine transaminase, U/L	67 (7–911)	65 (10–911)	71 (7–646)	0.65
γ‐glutamyl transferase, U/L	71 (11–1982)	67 (11–1288)	73 (13–1982)	0.31
Fasting blood glucose, mg/dL	105 (63–433)	105 (75–433)	104 (63–274)	0.11
Hemoglobin _A1c,_ %	5.7 (3.7–12.0)	5.8 (3.7–12.0)	5.6 (3.7–10.3)	0.13
IRI, μU/mL	11.4 (0.4–123.6)	11.6 (3.3–123.6)	11.4 (0.4–119.0)	0.13
Triglycerides, mg/dL	137 (21–674)	133 (32–415)	143 (21–676)	0.08
Total cholesterol, mg/dL	196 (65–482)	190 (65–328)	199 (67–482)	0.20
Platelet count, ×10^4^/μL	20.0 (3.7–112.0)	19.9 (3.7–96.0)	20.2 (3.9–112.0)	0.60
Prothrombin time, %	94.6 (9.6–110.0)	92.7 (9.6–106.0)	96.0 (11.3–110.0)	0.95
AFP, ng/mL	3 (1–119, 130)	4 (1–119, 130)	3 (1–22,699)	0.28
FIB‐4 index	1.61 (0.23–22.37)	1.81 (0.26–14.32)	1.46 (0.23–22.37)	0.83
Genotyping				
*PNPLA3* (GG, GC, CC)	160/169/58	84/88/35	76/81/23	0.52
Pathological findings of liver^a^				
Fibrosis < F3, ≥F3 (Percentage of advanced fibrosis ≥ F3, %)	193 vs. 111 (36.5%)	96 vs. 56 (36.8%)	97 vs. 55 (36.2%)	0.91
Steatosis < S2, ≥ S2 (Percentage of severe steatosis [≥ S2])	51 vs. 248 (82.9%)	34 vs. 116 (77.3%)	17 vs. 132 (88.6%)	<0.01
Activity < A2, ≥ A2 (Percentage of severe inflammation [≥ A2])	43 vs. 256 (85.6%)	22 vs. 128 (85.3%)	21 vs. 127 (85.8%)	0.91

Abbreviations: AFP, alpha‐fetoprotein; BMI, body mass index; CVD, cardiovascular disease; FIB‐4; fibrosis‐4; FLD, fatty liver disease; HCC, hepatocellular carcinoma; *HSD17B13*, 17‐beta dehydrogenase 13; IRI, immunoreactive insulin; NAFLD, nonalcoholic fatty liver disease; *PNPLA3*, patatin‐like phospholipase 3.

^a^ Liver biopsy was performed in 304 patients.

There were no significant differences in the percentage of liver tissue with severe fibrosis (F ≥ 3) or severe inflammation (A ≥ 2) among patients with various *HSD17B13* polymorphisms. Severe steatosis was more common among patients with *HSD17B13* variants than those with wild‐type polymorphism (S ≥ 2, 77.3% vs. 88.6%, *p* < 0.01).

### 3.2 HCC development according to gene polymorphisms and complications

**FIGURE 1:**
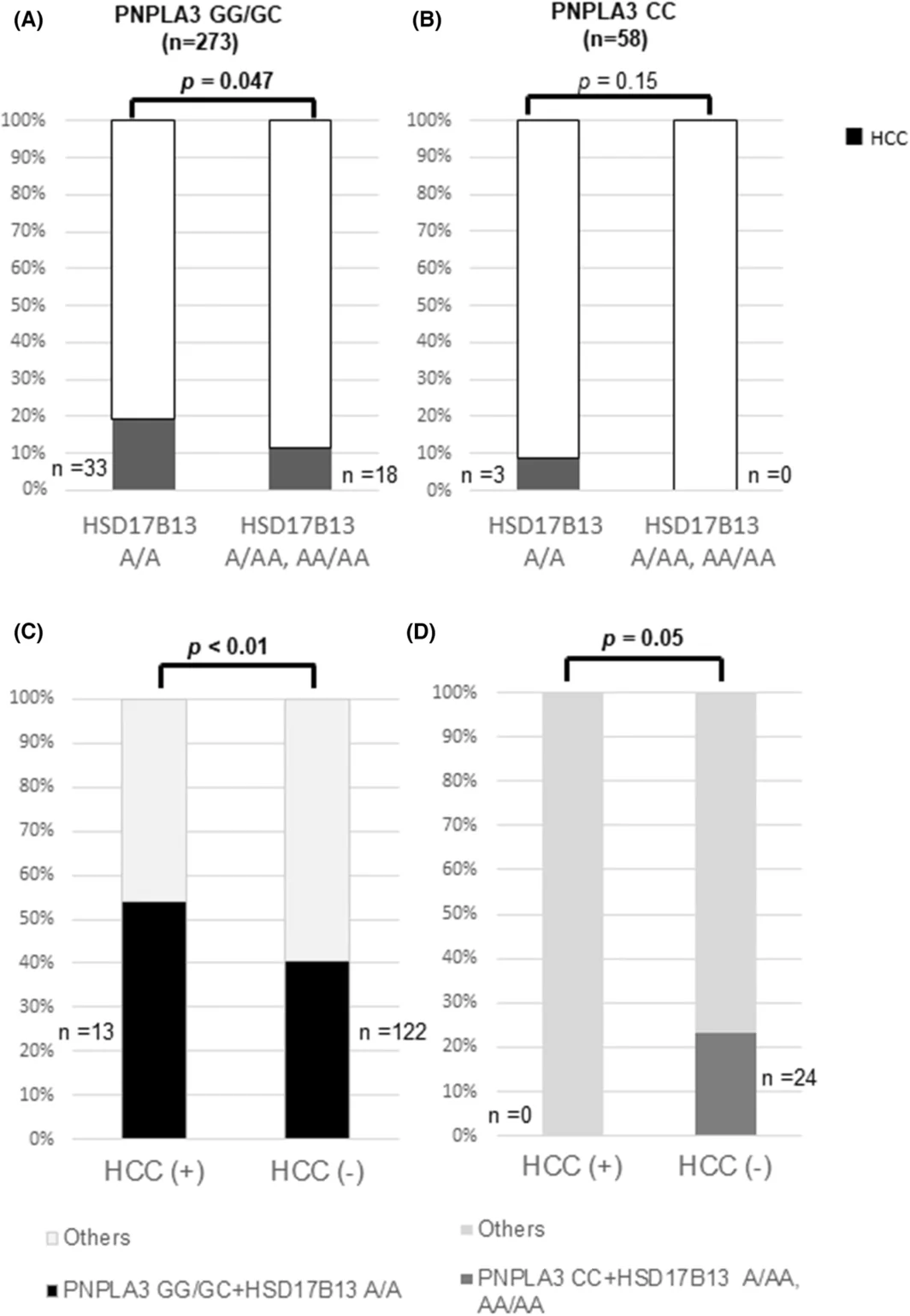
HCC complications stratified according to genetic factors. HCC incidence by (A) *PNPLA3* GG/GC and (B) *PNPLA3* CC according to the *HSD17B13* SNP, (C) combinations of risk SNPs, and (D) combinations of non‐risk SNPs. HCC was frequently observed in patients with *HSD17B13* A/A in *PNPLA3* GG/GC (A, *p* = 0.047). Among cases with *PNPLA3* CC, no HCC was observed among those with *HSD17B13* variants (B, *p* = 0.15). The incidence of HCC was significantly increased in patients with *PNPLA3* GG/GC and *HSD17B13* A/A (C, *p* < 0.01). Conversely, there were no HCC cases among patients with *PNPLA3* CC and *HSD17B13* variants (D, *p* = 0.05). HCC, hepatocellular carcinoma; *HSD17B13*, 17‐beta dehydrogenase 13; *PNPLA3*, patatin‐like phospholipase 3; SNP, single‐nucleotide polymorphism.

HCC was common in patients with *HSD17B13* A/A and *PNPLA3* GG/GC (Figure 1A, *p* = 0.047). Among patients with *PNPLA3* CC, no cases of HCC developed in those with *HSD17B13* variants (Figure 1B, *p* = 0.15). In combination with high‐risk SNPs, *PNPLA3* GG/GC and *HSD17B13* AA were associated with a significantly increased risk of HCC (Figure 1C, *p* < 0.01). Conversely, there were no HCC cases in patients with *PNPLA3* CC or *HSD17B13* variants (Figure 1D, *p* = 0.05).

**FIGURE 2:**
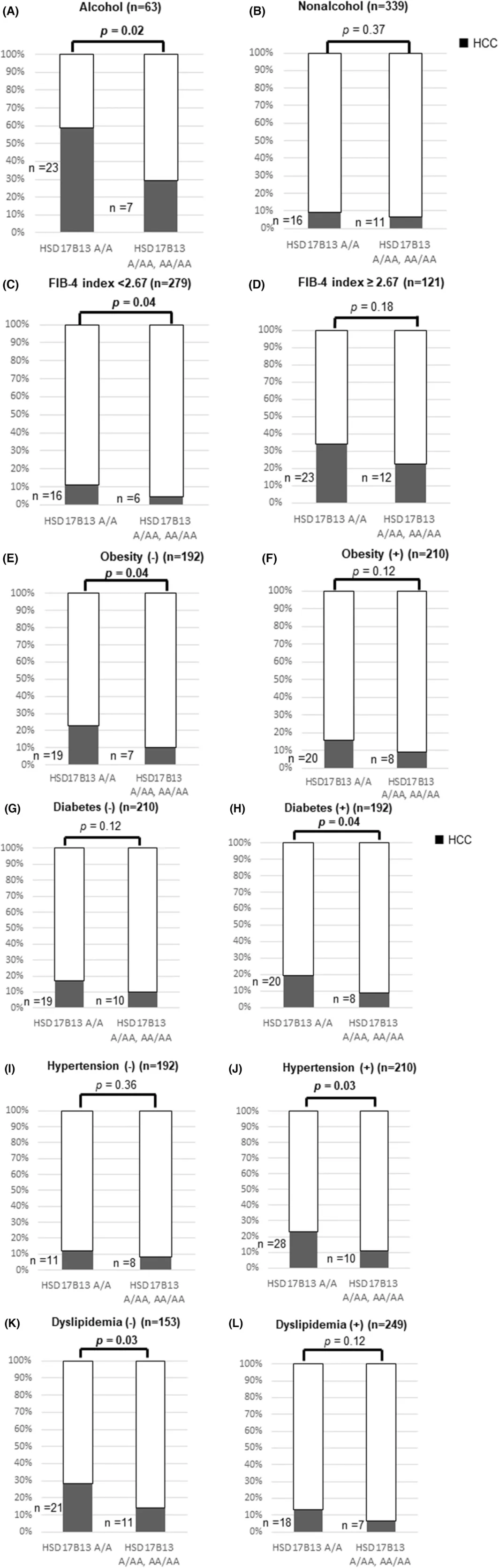
HCC incidence stratified by (A) alcohol‐related and (B) nonalcohol‐related pathology, and the presence and absence of (C, D) liver fibrosis, (E, F) obesity, (G, H) diabetes, (I, J) hypertension, and (K, L) dyslipidemia, according to *HSD17B13* SNP status. In patients without the *HSD17B13* A/A genotype, HCC incidence was significantly reduced among those with alcohol‐related FLD (A, *p* = 0.02), but not among those with nonalcoholic FLD (B, *p* = 0.37). HCC was more common in patients with *HSD17B13* A/A compared to *HSD17B13* variants and a FIB‐4 index <2.67 (C, *p* = 0.04), normal weight (E, *p* = 0.04), and normal lipid level (K, *p* = 0.03). However, there were no such significant differences among those with a FIB‐4 index ≥2.67 (D, *p* = 0.18, D), obesity (F, *p* = 0.12), or dyslipidemia (L, *p* = 0.12). The incidence of HCC was increased in patients with diabetes (H, *p* = 0.04) and hypertension (J, *p* = 0.03) among those with the *HSD17B13* A/A genotype compared to those with *HSD17B13* variants (G, *p* = 0.12, and I, *p* = 0.36, respectively). FIB‐4, fibrosis‐4; HCC, hepatocellular carcinoma; *HSD17B13*, 17‐beta dehydrogenase 13; *PNPLA3*, patatin‐like phospholipase 3.

In patients with *HSD17B13* variants, the HCC incidence was significantly reduced in alcohol‐related FLD (HSD17B13 variants, OR = 0.29, 95% CI = 0.10–0.85, *p* = 0.02, Figure 2A). However, the risk of HCC was not increased in NAFLD (*p* = 0.37, Figure 2B). After stratification according to the FIB‐4 index, HCC was more common in *HSD17B13* A/A than *HSD17B13* variants among those with a FIB‐4 index <2.67 (*p* = 0.04, Figure 2C), but not among those with a FIB‐4 index ≥2.67 (OR = 0.37, 95% CI = 0.14–0.98, *p* = 0.04, Figure 2D). The incidence of HCC was reduced in non‐obese patients with *HSD17B13* variants (*p* = 0.04, Figure 2E), but not in obese patients (*p* = 0.12, Figure 2F). The incidence of HCC was increased in patients with the *HSD17B13* A/A genotype among diabetic patients (*p* = 0.04, Figure 2H). HCC was more common in patients with the *HSD17B13* A/A genotype (*p* = 0.03, Figure 2J) than those with *HSD17B13* variants in hypertensive patients. However, there were no significant differences in HCC incidence among patients without complications of diabetes or hypertension (*p* = 0.12, Figure 2G and *p* =0.36, Figure 2I, respectively). The incidence of HCC was increased in patients with *HSD17B13* A/A in the absence of dyslipidemia (*p* = 0.03, Figure 2K). Furthermore, there were no significant differences in the risk of HCC development in patients with dyslipidemia stratified according to *HSD17B13* polymorphisms (*p* = 0.12, Figure 2L).

### 3.3 Multivariate analysis of HCC risk

To investigate whether these SNPs influence HCC development, a multivariate analysis was performed. In dataset 1, multivariate analysis identified risk factors of FLD–HCC including male sex (OR = 28.57, 95% CI = 3.636–200.000), age (OR = 1.08, 95% CI = 1.024–1.140), fibrosis (OR = 5.07, 95% CI = 1.954–13.159), and inflammation (activity) (OR = 0.13, 95% CI = 0.033–0.526) (all *p* < 0.01) (Table 2). In dataset 2, multivariate analysis revealed that the independent risk factors for HCC complications were male sex (OR = 5.24, 95% CI = 1.890–14.493), age (OR = 1.11, 95% CI = 1.061–1.162, *p* < 0.01), hypertension (OR = 5.42, 95% CI = 1.353–21.698, *p* = 0.02), and dyslipidemia (OR = 0.36, 95% CI = 0.132–0.977, p = 0.045) (Table 2). In the patients with new HCC development, the risk factors of HCC were evaluated by Cox proportional hazard model for dataset 2. Because new HCC evaluated by pathological examination was low incidence in our cohort, dataset 2 was used. Complication of diabetes (OR = 12.38, 95% CI =1.298–118.038, *p* = 0.03) and non‐complication of dyslipidemia (OR = 0.11, 95% CI = 0.024–0.544, *p* < 0.01) were observed as the risk factors. The multivariate analysis did not identify the SNPs as risk factors of HCC.

**TABLE 2:** HCC risk factors according to multivariate analysis.

	Odds ratio	95% confidence interval	*p*‐value
(A). Dataset 1: age, sex, BMI, pathological fibrosis, inflammation, and steatosis, lifestyle‐related diseases, and *PNPLA3* and *HSD17B13* SNPs			
Sex (male)	28.57	3.636–200.000	<0.01
Age	1.08	1.024–1.140	<0.01
Fibrosis	5.07	1.954–13.159	<0.01
Inflammation	0.13	0.033–0.526	<0.01
(B). Dataset 2; Age, sex, BMI, FIB‐4 index, lifestyle‐related diseases, and *PNPLA3* and *HSD17B13* SNPs			
Sex (male)	5.24	1.890–14.493	<0.01
Age	1.11	1.061–1.162	<0.01
Hypertension	5.42	1.353–21.698	0.02
Dyslipidemia	0.36	0.132–0.977	0.045

Abbreviations: BMI, body mass index; FIB‐4; fibrosis‐4; HCC, hepatocellular carcinoma; *HSD17B13*, 17‐beta dehydrogenase 13; *PNPLA3*, patatin‐like phospholipase 3.

### 3.4 New‐onset HCC development stratified according to *PNPLA3* and *HSD17B13* polymorphisms

**FIGURE 3:**
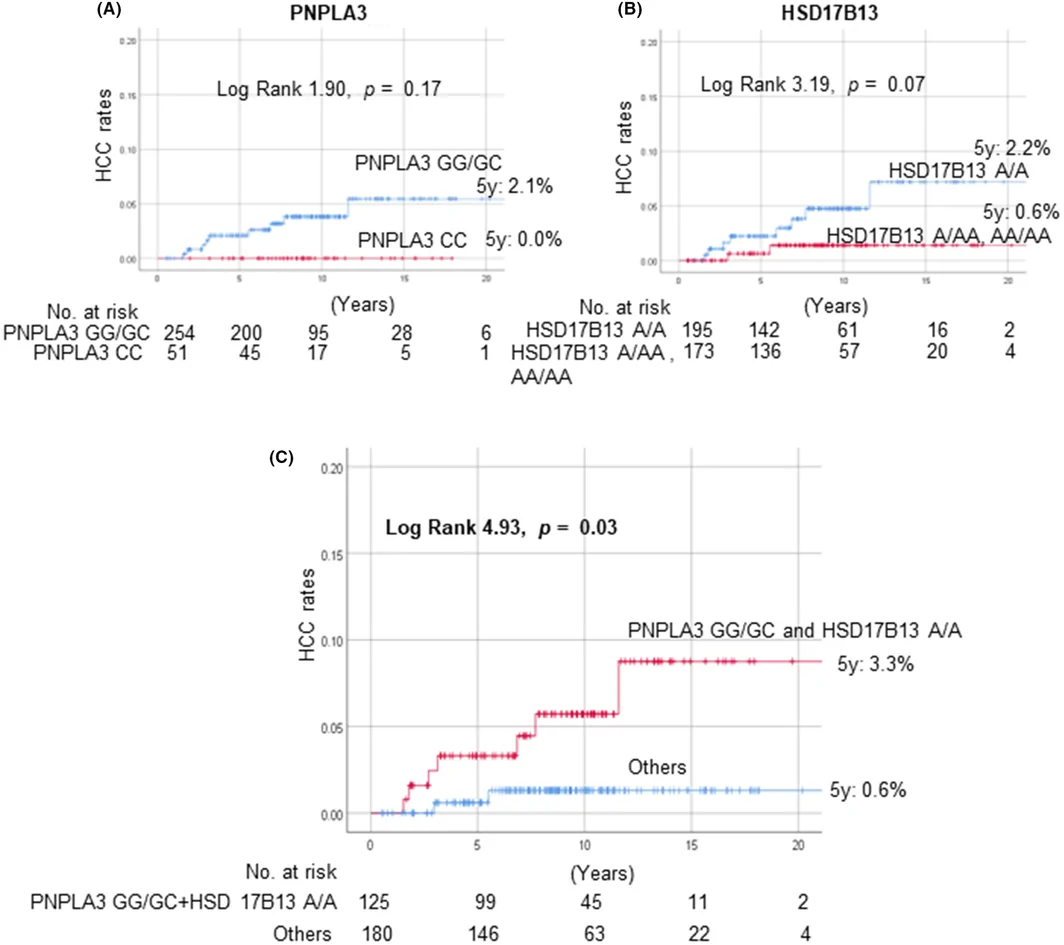
New‐onset HCC rate according to (A) *PNPLA3*, (B) *HSD17B13*, and (C) both *PNPLA3* + *HSD17B13* SNP status, as determined by Kaplan–Meier curves. There were no significant differences and a trend toward an increased risk of HCC development in patients with the *PNPLA3* genotype (A, *p* = 0.17) and *HSD17B13* genotype (B, *p* = 0.07), respectively. The incidence of de novo HCC was significantly increased in patients with *PNPLA3* GG/GC and *HSD17B13* A/A (log‐rank = 4.93, *p* = 0.03). HCC, hepatocellular carcinoma; *HSD17B13*, 17‐beta dehydrogenase 13; *PNPLA3*, patatin‐like phospholipase 3; SNP, single‐nucleotide polymorphism.

Eleven new cases of HCC developed over 8.1 (0.5–25.1) years of the observation period (*n* = 370). Among patients with the *PNPLA3* CC genotype, none developed HCC in 5 years, whereas HCC was observed in 2.1% of cases with PNPLA3 GG/GC after 5 years (*p* = 0.17, Figure 3A). HCC developed in 2.2% of patients with *HSD17B13* A/A and 0.6% of patients with *HSD17B13* variants over 5 years (*p* = 0.07, Figure 3B). In patients with both *PNPLA3* GG/GC and *HSD17B13* A/A, HCC was significantly more common than in patients with other SNPs (*p* = 0.03, Figure 3C).

## 4 DISCUSSION

This study assessed the rate of HCC development in FLD patients according to the genetic polymorphisms of *PNPLA3* and *HSD17B13*. The genetic background of FLD may be associated with increased or decreased risk of HCC. Previous studies have shown that *PNPLA3* GG/GC is significantly associated with an increased risk of HCC development in FLD,^3^ whereas *HSD17B13* variants are associated with a reduced risk of HCC onset.^10^ Our cohort study showed similar results; the combination of high‐risk alleles of *PNPLA3* and *HSD17B13* should prompt frequent screening for HCC. Among patients with *HSD17B13* variants, the HCC risk was reduced in those with a FIB‐4 index <2.67, normal body weight, and normal lipid levels; conversely, the HCC risk was increased in patients with diabetes mellitus and hypertension among those with the *HSD17B13* A/A genotype. Liver fibrosis and obesity may be more strong factors for carcinogenesis than suppression by genetic factors.

Gellert‐Kristensen et al.^17^ demonstrated that *HSD17B13* rs72613567: TA is associated with decreased alanine aminotransferase levels in the Danish general population. Although there were no significant abnormalities in liver enzymes in the present study, the serum triglyceride level was likely to be increased in patients with *HSD17B13* variants. Rotroff et al.^18^ demonstrated that *HSD17B13* variants are associated with higher levels of triglycerides and high‐density lipoprotein levels in White populations. Our data showed that the incidence rates of CVD and hypertension were significantly reduced in patients with *HSD17B13* variants. No evidence is available to confirm whether or not *HSD17B13* variants protect against CVD or hypertension.

In terms of the effect of *HSD17B13* on liver pathology, the rs72613567:TA variant is associated with a reduced risk of NASH but not steatosis.^9^ Ma et al.^19^ demonstrated that *HSD17B13* functions as a retinol dehydrogenase and is associated with histological features of NAFLD. However, it does not affect hepatocyte lipid content directly. In our study, *HSD17B13* variants increased the pathological fat content and frequency of S ≥ 2 compared to the *HSD17B13* A/A genotype. In Japanese patients with NAFLD, the *HSD17B13* rs6834314 G allele was associated with a significantly lower prevalence of severe inflammation and ballooning, as well as a higher prevalence of advanced steatosis.^20^ In carriers of this allele, the effects of the *PNPLA3* rs738409 GG genotype on advanced hepatic fibrosis are attenuated.^20^ However, the prevalence of advanced fibrosis and severe inflammation was not significantly increased in those with *HSD17B13* variants in this study.

Yang et al.^10^ demonstrated the protective effects of the *HSD17B13* rs72613567: TA allele on HCC development. Among 3315 patients, HCC was reduced in those with ALD (OR = 0.64).^10^ Abul‐Husn et al.^9^ showed that the minor allele of *HSD17B13* rs72613567 reduced the risk of ALD by 53% and of NAFLD by 30%. Our study also showed that the risk of HCC development was reduced with this allele, particularly in patients with ALD. The incidence of HCC was also reduced in patients with a FIB‐4 index <2.67, normal weight, and normal lipid levels. Among patients with diabetes and hypertension, *HSD17B13* A/A carriers have a higher risk of HCC. Therefore, *HSD17B13* may be an independent protective factor against HCC development, in association with the low‐risk factors of fibrosis and obesity. Conversely, among patients with high‐risk factors of HCC, such as diabetes and hypertension, the *HSD17B13* A/A genotype was associated with an increased risk of HCC. In other words, the incidence of HCC was reduced in patients with *HSD17B13* variants who had diabetes and hypertension, although there was no significant difference among patients without diabetes and hypertension. It is possible that our results were due to the fact that the incidence of HCC was not reduced in NAFLD patients with *HSD17B13* variants (data not shown). The incidence of HCC development is low in NAFLD patients without diabetes or hypertension and can be affected by the genetic background.

In a previous study, multivariate analysis showed that age, male sex, diabetes, platelet count, gamma‐glutamyltransferase and albumin levels, and the 7‐SNP genetic risk score, including *PNPLA3* and *HSD17B13*, are independent risk factors of HCC development.^21^ We performed a multivariate analysis of two independent datasets to determine the risk factors of HCC. Dataset 1 included pathological fibrosis, whereas dataset 2 included the FIB‐4 index. Although our study did not identify genetic polymorphism as an independent risk factor of FLD‐HCC, age, male sex, hypertension, and fibrosis were major risk factors of HCC. Low inflammation and normal lipid level may result from liver cirrhosis. These factors are more strongly related to carcinogenesis in FLD compared to genetic factors.

In the analysis of new‐onset HCC, *HSD17B13* variants were associated with a decreased 5‐year incidence rate of HCC (0.6%, *p* = 0.07); however, the risk was not increased with *PNPLA3*. The combination of high‐risk alleles of *PNPLA3* GG/GC and *HSD17B13* A/A was associated with a significantly higher risk of HCC compared to other SNPs (*p* = 0.03), indicating that it is a good screening tool for HCC development in FLD.

This study had several limitations. First, this was a single‐center observational study. Second, the number of new cases of HCC was small, and evaluation of HCC development was mainly in the patients with HCC complications, it was not enough in the patients with new HCC. However, it was not particularly low in carcinogenesis of NAFLD in our institute, in a nationwide survey conducted in Japan, the annual incidence of HCC was 0.11%.^2^ The incidence of HCC among patients with non‐cirrhotic NAFLD is reported approximately from 0.1 to 1.3 per 1000 patient‐years.^22^ Third, only a small number of ALD patients were included. Fourth, in cirrhosis, synthesis of lipid was decreased and low hemoglobin levels due to pancytopenia or bleeding may mask dyslipidemia or diabetes. Finally, we could not separately assess the influence of therapeutic drugs and lifestyle modification including body weight reduction and nutrition of the patients. Future studies should include larger sample sizes.

## 5 CONCLUSIONS

We identified genetic risk and protective factors of HCC development among Japanese patients with FLD according to their metabolic risk. These genetic polymorphisms can be used to screen for HCC development in FLD.

## AUTHOR CONTRIBUTIONS

**Tomomi Kogiso:** Data curation (equal); investigation (equal); writing – original draft (equal); writing – review and editing (equal). **Yuri Ogasawara:** Data curation (equal). **Kentaro Horiuchi:** Data curation (equal). **Makiko Taniai:** Data curation (equal). **Katsutoshi Tokushige:** Supervision (equal); writing – review and editing (equal).

## CONFLICT OF INTEREST STATEMENT

KT is the recipient of research funding from Sumitomo Dainippon Pharma Co., Ltd., Astellas Pharma Inc., Eisai Co., Ltd., Taiho Pharmaceutical Co., Ltd., Chugai Pharmaceutical Co., Ltd., Daiichi Sankyo Pharmaceutical Co., Ltd., AbbVie GK, Takeda Pharmaceutical Co. Ltd., Asahi Kasei Corporation. Ajinomoto Co., Inc., and Otsuka Pharmaceutical Co., Ltd.

## ETHICS STATEMENT

This study was conducted in accordance with the Declaration of Helsinki and the ethical guidelines of the Tokyo Women's Medical University Hospital (Tokyo, Japan). The Institutional Review Board of Tokyo Women's Medical University Hospital approved the study protocol (No. 323). *Approval of the research protocol*: No. 323.

## INFORMED CONSENT

Informed consent was obtained from the study participants.

## Data Availability

The datasets used and/or analyzed in this study are available from the corresponding author upon reasonable request.
